# Evaluation of PD-L1 Expression Level in Patients With Non-Small Cell Lung Cancer by ^18^F-FDG PET/CT Radiomics and Clinicopathological Characteristics

**DOI:** 10.3389/fonc.2021.789014

**Published:** 2021-12-16

**Authors:** Jihui Li, Shushan Ge, Shibiao Sang, Chunhong Hu, Shengming Deng

**Affiliations:** ^1^ Department of Nuclear Medicine, The First Affiliated Hospital of Soochow University, Suzhou, China; ^2^ Department of Radiology, The First Affiliated Hospital of Soochow University, Suzhou, China; ^3^ Department of Nuclear Medicine, Suqian First Hospital, Suqian, China; ^4^ State Key Laboratory of Radiation Medicine and Protection, Soochow University, Suzhou, China

**Keywords:** non-small cell lung cancer, PD-L1 immunotherapy, radiomics, ^18^F-FDG PET/CT, clinicopathological

## Abstract

**Purpose:**

In the present study, we aimed to evaluate the expression of programmed death-ligand 1 (PD-L1) in patients with non-small cell lung cancer (NSCLC) by radiomic features of ^18^F-FDG PET/CT and clinicopathological characteristics.

**Methods:**

A total 255 NSCLC patients (training cohort: n = 170; validation cohort: n = 85) were retrospectively enrolled in the present study. A total of 80 radiomic features were extracted from pretreatment ^18^F-FDG PET/CT images. Clinicopathologic features were compared between the two cohorts. The least absolute shrinkage and selection operator (LASSO) regression was used to select the most useful prognostic features in the training cohort. Radiomics signature and clinicopathologic risk factors were incorporated to develop a prediction model by using multivariable logistic regression analysis. The receiver operating characteristic (ROC) curve was used to assess the prognostic factors.

**Results:**

A total of 80 radiomic features were extracted in the training dataset. In the univariate analysis, the expression of PD-L1 in lung tumors was significantly correlated with the radiomic signature, histologic type, Ki-67, SUV_max_, MTV, and TLG (p< 0.05, respectively). However, the expression of PD-L1 was not correlated with age, TNM stage, and history of smoking (p> 0.05). Moreover, the prediction model for PD-L1 expression level over 1% and 50% that combined the radiomic signature and clinicopathologic features resulted in an area under the curve (AUC) of 0.762 and 0.814, respectively.

**Conclusions:**

A prediction model based on PET/CT images and clinicopathological characteristics provided a novel strategy for clinicians to screen the NSCLC patients who could benefit from the anti-PD-L1 immunotherapy.

## Introduction

Lung cancer is a malignant tumor with the highest morbidity and mortality in the world, and its average 5-year survival rate is only 15% (1). Non-small cell lung cancer (NSCLC) accounts for 80% ~ 85% of all lung cancer cases (2). Early diagnosis and treatment play a key role in improving the 5-year survival rate of lung cancer. In recent years, with the further study of tumor immune microenvironment, immunotherapy has developed rapidly, attracted more and more oncologists’ attention, and become an important research field of tumor therapy, including lung cancer (3). The immunotherapy against programmed cell death protein 1 (programmed death-1, PD-1) and its ligand 1 (programmed death ligand-1, PD-L1) has been used in NSCLC, and good results have been achieved in patients, especially in individuals with high expression of PD-L1 (4, 5).

At present, the expression of PD-L1 in clinical practice is usually detected through the “gold standard” of immunohistochemistry (IHC) (6, 7). It is difficult to obtain a clear expression level of PD-L1 in high-quality tissue samples. Moreover, small tissue samples, such as biopsies, may not be representative of tumors because of the intratumor heterogeneity (8). Some studies have demonstrated that PD-1/PD-L1 inhibitors can benefit patients who have failed first-line chemotherapy when the PD-L1 expression rate is higher than 1%. Moreover, PD-1/PD-L1 inhibitors can even be used as a preferred treatment for patients when the PD-L1 expression exceeds 50% (9). Therefore, it is urgently necessary to find a new approach to assess the expression level of PD-L1.

Radiomics is an emerging field with great development potential, which was first proposed by Dutch scholar Lambin in 2012 (10). In recent years, image omics has developed rapidly, and optimistic results have been achieved in the diagnosis and differential diagnosis of diseases, tumor staging and grading, gene-phenotype prediction, treatment plan decision-making, efficacy evaluation, and prognostic prediction (11–13). In particular, it shows great superiority in lung tumors (14). However, biopsies capture heterogeneity within only a small portion of a tumor and usually at just a single anatomic site, while radiomics captures heterogeneity across the entire tumor.

Combination of functional-metabolic and morphological imaging and F18-fluorodeoxyglucose-positron emission tomography/computed tomography (^18^F-FDG PET/CT) is the most advanced non-invasive imaging technology at present, which can reflect the glucose uptake level of tissues to a certain extent. It has important application value in the diagnosis, staging, curative effect, and prognostic evaluation of lung cancer (15–17). Several studies have reported the role of radiomics in various malignancies (18, 19). However, research using radiomics based on ^18^F-FDG PET/CT in combination with clinical risk factors for NSCLC is relatively limited.

In the present study, we aimed to develop a prediction model that incorporated both the radiomic signature and clinicopathologic risk factors for individual prediction of PD-L1 expression in NSCLC patients. Our findings could be helpful to identify the patients who could benefit from the immunotherapy.

## Materials and Methods

### Patients

A retrospective study consisting of NSCLC patients who underwent a combined imaging protocol of ^18^F-FDG PET/CT between January 2019 and March 2021 was conducted. Ethical approval was obtained for this retrospective analysis, and the informed consent requirement was waived. A total of 255 patients were randomly divided into the training (n = 170) and validation (n = 85) cohorts following a ratio of 7:3 (20). Inclusion criteria were as follows: (a) patients who underwent biopsy or surgery of lung tumor; (b) patients with IHC examination of PD-L1 performed; (c) histological type and grade were pathologically proven; and (d) standard ^18^F-FDG PET/CT was performed before biopsy or surgery. Exclusion criteria were as follows: (a) therapy (radiotherapy, chemotherapy, or chemoradiotherapy) was performed before ^18^F-FDG PET/CT and IHC; (b) patients with unknown histological grade; (c) the size of the primary lesion was too small for segmentation; and (d) patients with other types of cancers or with incomplete clinical and imaging datasets.

### Detection of PD-L1 Expression

The biopsy and surgery specimens of lung tumors through hematoxylin-eosin (H&E) staining were pathologically examined to confirm the histologic type and grade under the microscope. Furthermore, the expression of PD-L1 was determined through the IHC assay in our study. PD-L1 test kit (22C3 pharmDx) was obtained from the Dako company. The back-to-back interpretation of PD-L1 expression was performed by pathologists, and further reanalysis would be implemented when there was an inconsistency compared with previous results. The data of patients were divided with a PD-L1 cutoff value of 1% and 50%.

### PET/CT Image Acquisition and Reconstruction

Patients were recommended to fast for at least 4 h before the FDG-PET/CT scan (4.07-5.55 MBq/kg). Blood glucose levels were maintained at less than 11 mmol/L. A whole-body scan was acquired at 60 ± 10 min after intravenous injection of ^18^F-FDG using an integrated PET/CT scanner (Discovery STE; General Electric Medical Systems, Milwaukee WI, USA). First, low-dose CT images were performed, with parameters as follows: 140 kV, 120 mA, transaxial field of view (FOV) of 70 cm, pitch of 1.75, rotation time of 0.8 s, and slice thickness of 3.75 mm, followed by PET images, with 2-3 min per bed position and 7-8 bed positions per patient.

### Feature Segmentation and Extraction

**Figure 1:**
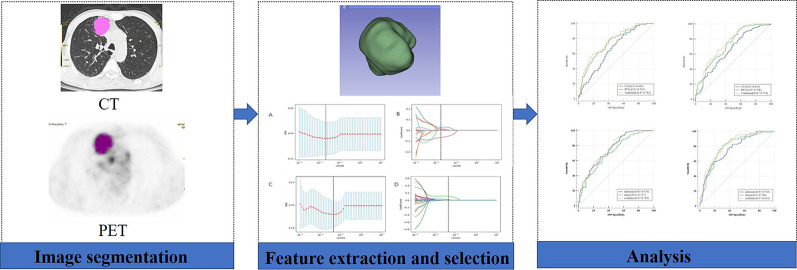
Workflow of the radiomic analysis. A 58-year-old man underwent ^18^F-FDG PET/CT for staging workup of NSCLC patients with a SUV_max_ of 11.4. The VOI of the lesion was manually delineated, and 41% of SUV_max_ was applied as a threshold to optimize the VOI.

**Figure 2:**
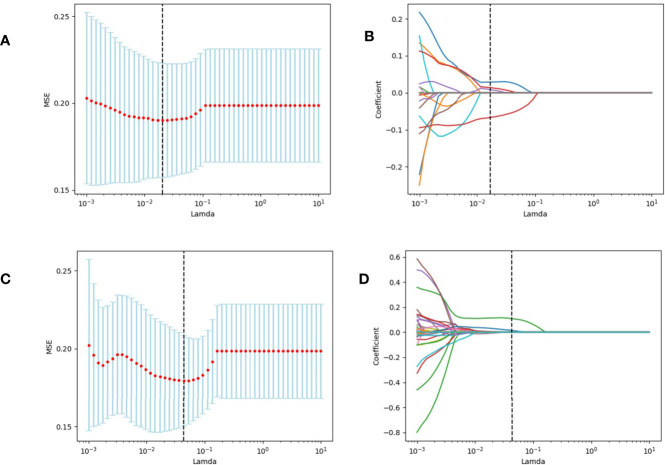
We used two feature selection methods, maximum relevancy and minimum redundancy (MRMR) and the LASSO to select the radiomic feature of CT and PET. At first, MRMR was performed to eliminate the redundant and irrelevant features. Then LASSO was conducted to choose the optimized subset of features to construct the final model. **(A, C)** Tuning parameter Lambda (λ) selection in the LASSO model used 10-fold cross-validation *via* minimum criteria. **(B, D)** LASSO coefficient profiles of the retained features. A vertical line was drawn at the value selected using 10-fold cross-validation.

Tumor segmentation was performed to select primary lesions of NSCLC cases after image acquisition. **Figure 1** shows the workflow of radiomics analysis in this study. PET and CT images of the DICOM format were transferred to LIFEx freeware and automatically fused by the freeware. The LIFEx freeware was used to do quantitative PET/CT analyses (v7.0.0 https://www.lifexsoft.org/) (21). Two experienced nuclear medicine physicians manually segmented the three-dimensional volume of interest (VOI) on each slice, and a threshold of 41% of the maximum standardized uptake value (SUV_max_) was used to define VOI, including metabolic tumor volume (MTV) and total focal glycolysis (TLG) of lesions. TLG is the MTV multiplied by the mean SUV of the tumor. The voxel size for spatial resampling was 2 × 2 × 2 mm. For CT data, intensity discretization was done with 400 gray levels and absolute scale boundaries between -1,000 and 3,000 HU, whereas for PET data, it was done with 64 bins between 0 and 20. The intraclass correlation coefficient (ICC) was used to determine the repeatability/reproducibility of features in our research, and ICC >0.75 was selected. Subsequently, the least absolute shrinkage and selection operator (LASSO) COX regression model was used to select the most useful prognostic features with 10-fold cross-validation for selecting the parameter Lambda in the training cohort (**Figure 2**) (22, 23).

### Prediction Model

Univariable logistic regression analysis began with the following clinical candidate predictors: age, gender, tumor location, histology type and grade, CEA level, smoking history, Ki-67. The radiomic signature and clinicopathologic risk factors with statistically significant differences were incorporated to develop a prediction model by using multivariable logistic regression analysis in a training cohort consisting of 170 consecutive patients, and the receiver operating characteristic (ROC) curve and the corresponding area under the curve (AUC) were reckoned for the prediction model in the training cohort and validation cohort, respectively.

### Statistical Analysis

All statistical analyses were operated with SPSS software version 26.0 (SPSS Inc., Chicago, IL, USA) and python 3.8.0 (https://www.python.org). The differences in patients’ characteristics between the training and validation cohorts were compared using the Chi-square test. The spearman rank-order correlation was calculated to analyze the relevance between the expression of PD-L1 and selected features. AUC of the ROC was calculated to evaluate the performance of our prediction model. A p < 0.05 was considered statistically significant.

## Results

### Patients’ Characteristics

**Figure 3:**
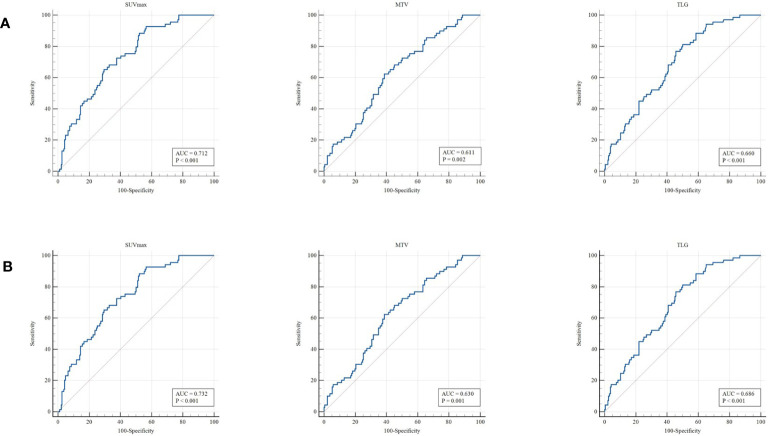
ROC curve for the determination of the most discriminative cutoff points for SUV_max_, MTV, and TLG in primary tumors. The optimal cutoff values of SUVmax, MTV, and TLG for the PD-L1 1% group **(A)** were 5.21, 123.94, and 216.62, respectively. The optimal cutoff values of SUV_max_, MTV, and TLG for the PD-L1 50% group **(B)** were 6.82, 137.57, and 191.68, respectively.

**Table 1** summarizes the clinical characteristics of patients in the training and validation cohorts. A total of 255 patients were enrolled in this study. Among the patients selected, 188 cases (73.7%) were adenocarcinoma, and 67 (26.3%) were squamous cell carcinoma. We demonstrated that several clinicopathologic characteristics might be associated with the expression of PD-L1. Of these patients, there were not any statistically significant differences in the clinical characteristics between the training and validation cohorts (**Table 1**). In our univariate analysis, the expression of PD-L1 was significantly correlated with gender, histologic type, tumor location, and Ki-67 (p< 0.05, respectively). However, it was not correlated with age, TNM stage, and history of smoking (p> 0.05, respectively) (**Table 2**). Based on the ROC analysis, the optimal cutoff values of SUV_max_, MTV, and TLG for the PD-L1 1% group were 5.21, 123.94, and 216.62, respectively. Moreover, the optimal cutoff values of SUV_max_, MTV, and TLG for the PD-L1 50% group were 6.82, 137.57, and 191.68, respectively (**Figure 3**).

**Table 1:** Characteristics of the training and validation cohorts.

Characteristics	Total (n = 255)	Training (n = 170)	Validation (n = 85)	t/χ^2^	p
Sex				0.009	0.925
Male	170	113	57		
Female	85	57	28		
Age, median ± SD, years	64.22 ± 9.51	64 ± 9.07	64.66 ± 10.37	-.0659	0.603
Tumor location				0.133	0.715
Left lung	97	66	31		
Right lung	158	104	54		
Histologic type, No. (%)				0.04	0.841
Squamous cell carcinoma	67	44	23		
Adenocarcinoma	188	126	62		
TNM stage, No. (%)				2.758	0.097
I-II	194	124	70		
III- IV	61	46	15		
Smoking history				0.314	0.575
Smoker	168	110	58		
Never	87	60	27		
Ki67					
<20%	68	39	29	3.62	0.057
≥20%	187	131	56		

**Table 2:** Characteristics of NSCLC patients with different PD-L1 expression levels.

Characteristics	PD-L1 < 1%(n = 101)	PD-L1≥1% (n = 154)	t/χ^2^	p	PD-L1 < 50% (n = 186)	PD-L1≥50% (n = 69)	t/χ^2^	p
Sex			3.967	0.046*			7.242	0.001*
Male	60	110			115	55		
Female	41	44			71	14		
Age, median ± SD, years	63.26 ± 9.46	64.85 ± 9.52			64.15 ± 9.44	64.41 ± 9.75	-0.255	0.849
Tumor location			2.867	0.09			7.996	0.005*
Left lung	32	65			66	38		
Right lung	69	89			120	31		
Histologic type, No. (%)			6.169	0.013*			4.842	0.028*
Squamous cell carcinoma	18	49			42	25		
Adenocarcinoma	83	105			144	44		
TNM stage, No. (%)			27.617	0.000*			2.634	0.105
I-II	86	82			128	40		
III- IV	15	72			58	29		
Smoking history			5.321	0.021*			1.108	0.292
Smoker	58	110			119	49		
Never	43	44			67	20		
Ki67			11.721	0.001*			5.564	0.018*
<20%	41	32			57	11		
≥20%	60	122			129	58		
SUV_max_			30.304	0.000*			29.694	0.000*
High	58	135			105	64		
Low	43	19			81	5		
MTV			10.924	0.001*			9.998	0.002*
High	37	89			72	42		
Low	64	65			114	27		
TLG			17.149	0.001*			20.118	0.000*
High	41	103			93	56		
Low	60	51			93	13		

*Statistically significant, p < 0.05.

### Feature Selection in the Training Cohort

A total of 80 radiomic features were extracted in the training dataset (**Table 3**). For the prediction of PD-L1 expression level over 1%, 18 features considered valuable for predicting the PD-L1 expression were extracted, including six features from the CT dataset and 12 features from the PET dataset. For the prediction of PD-L1 expression level over 50%, seven features considered valuable for predicting the PD-L1 expression were extracted, including four features from the CT dataset and three features from the PET dataset. The ICC of the radiomic features was all above 0.75.

**Table 3:** Radiomic parameters.

Conventional textural features	First-order textural features
SUVmin SUVmean SUVstd SUVmax SUVpeak* TLG*	HISTO_Skewness HISTO_Kurtosis HISTO_Entropy_log10 HISTO_Entropy_log2 HISTO_Energy SHAPE_Sphericity SHAPE_Compacity SHAPE_Volume (mL) SHAPE_Volume(vx)
**Higher-order textural features**	
**GLZLM**	**GLRLM**
GLZLM_SZE (Short-Zone Emphasis) GLZLM_LZE (Long-Zone Emphasis) GLZLM_LGZE (Low Gray-level Zone Emphasis) GLZLM_HGZE (High Gray-level Zone Emphasis) GLZLM_SZLGE (Short-Zone Low Gray-level Emphasis) GLZLM_SZHGE (Short-Zone High Gray-level Emphasis) GLZLM_LZLGE (Long-Zone Low Gray-level Emphasis) GLZLM_LZHGE (Long-Zone High Gray-level Emphasis) GLZLM_GLNU (Gray-Level Non-Uniformity for zone) GLZLM_ZLNU (Zone Length Non-Uniformity) GLZLM_ZP (Zone Percentage)	GLRLM_SRE (Short-Run Emphasis) GLRLM_LRE (Long-Run Emphasis) GLRLM_LGRE (Low Gray-level Run Emphasis) GLRLM_HGRE (High Gray-level Run Emphasis) GLRLM_SRLGE (Short-Run Low Gray-level Emphasis) GLRLM_SRHGE (Short-Run High Gray-level Emphasis) GLRLM_LRLGE (Long-Run Low Gray-level Emphasis) GLRLM_LRHGE (Long-Run High Gray-level Emphasis) GLRLM_GLNU (Gray-Level Non-Uniformity for run) GLRLM_RLNU (Run Length Non-Uniformity) GLRLM_RP (Run Percentage)
**GLCM**	**NGLDM**
GLCM_Homogeneity GLCM_Energy GLCM_Contrast GLCM_Correlation GLCM_Entropy_log10 GLCM_Entropy_log2 GLCM_Dissimilarity	NGLDM_Coarseness NGLDM_Contrast NGLDM_Busyness

*Calculated only for PET.

### Diagnostic Validation of Radiomic Signature and Clinical Features

**Figure 4:**
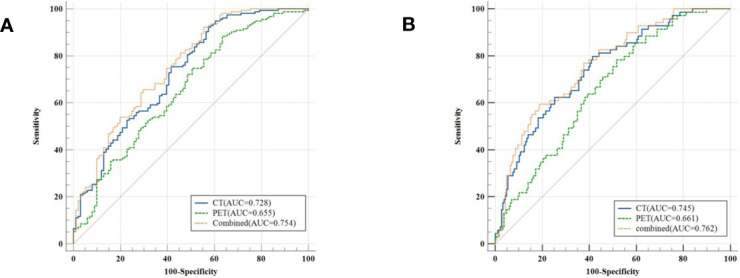
Classifiers’ performance on predicting the expression status of PD-L1 based on three models (CT, PET, and the combined model). Classifiers’ performance on predicting 1% level of PD-L1 **(A)**. Classifiers’ performance on predicting 50% level of PD-L1 **(B)**.

**Figure 5:**
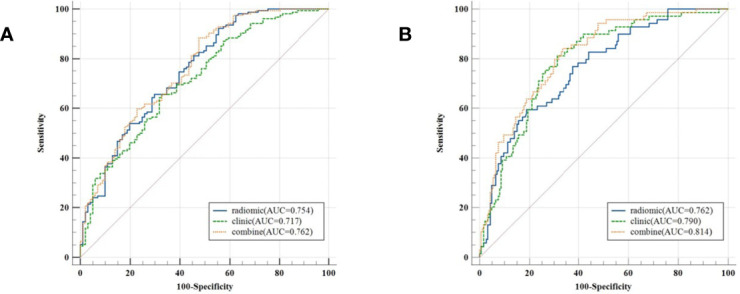
Classifiers’ performance on predicting the expression status of PD-L1 based on three models (radiomics, clinics, and the combined model). Classifiers’ performance on predicting 1% level of PD-L1 **(A)**. Classifiers’ performance on predicting 50% level of PD-L1 **(B)**.

**Figure 6:**
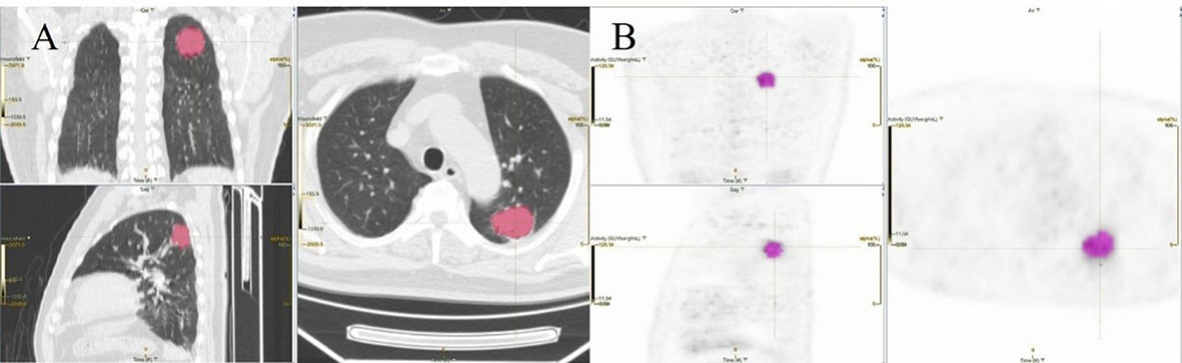
An example of segmentation coronary image, sagittal image, and transaxial images of CT **(A)** and PET **(B)** showing an example of VOI for measuring imaging features of NSCLC.

The model evaluation was conducted in the testing cohort. **Figure 4** shows the AUCs of ROC for the three models (CT, PET, and the combined model) in predicting the PD-L1 expression ≥ 1% and 50%. The AUC scores for predicting the PD-L1 expression over 1% were 0.655 (95% confidence interval [CI]: 0.593-0.713) and 0.728 (95% CI: 0.699-0.782) for features derived from CT and PET only, respectively, and it became 0.754 (95% CI: 0.696-0.805) for combined features. For the prediction of PD-L1 expression over 50%, the AUC scores were 0.661 (95% CI: 0.599-0.719), 0.745 (95% CI: 0.687-0.797), and 0.762 (95% CI: 0.705-0.813) for features derived from CT, PET, and combined model, respectively. **Figure 5** shows the AUCs of ROC for the three models (radiomics, clinics, and the combined model) in predicting PD-L1 expression ≥ 1% and 50%. Using LIFEx, the region of interest (ROI) was initially identified around the tumor outline on the CT and PET images (**Figure 6**).

For the prediction of PD-L1 expression over 1%, the AUC scores were 0.754 (95% CI: 0.696-0.805), 0.636 (95% CI: 0.574-0.695), and 0.757 (95% CI: 0.699-0.808) for features derived from radiomics, clinics, and combined model, respectively. For the prediction of PD-L1 expression over 50%, the AUC scores were 0.762 (95% CI: 0.705-0.813), 0.672 (95% CI: 0.611-0.730), and 0.814 (95% CI: 0.761-0.860) for features derived from radiomics, clinics, and combined model, respectively.

## Discussion

Our present study demonstrated that ^18^F-FDG PET/CT radiomic signature was useful for assessing the expression rate of PD-L1 through radiomic features in NSCLC patients. Radiomic signature successfully stratified patients according to the PD-L1 expression rate threshold of 1% and 50%. The combination of the radiomic signature and clinicopathologic risk factors presented a better diagnostic efficacy compared with the simple radiomic signature or clinical feature model.

Studies have shown that PD-L1 is highly expressed on the surface of a variety of tumor cells (including lung cancer) (24–27). PD-1/PD-L1 inhibitors can exert the immune effect of T cells against tumors in a variety of ways and inhibit tumor development. More and more clinical evidence supports the effect of PD1/PD-L1 inhibitors in the treatment of lung cancer (28). Varying degrees of survival benefit and delay of disease progression in patients with lung adenocarcinoma can be achieved no matter PD-1/PD-L1 inhibitors are used alone or in combination with chemotherapy and molecular targeted therapy. At present, IHC is the main method utilized in detecting the PD-L1 expression rate. Several preclinical PET studies have also demonstrated non-invasive imaging of PD-L1 expression in tumors (29, 30).

At present, radiomics has been widely used in lung cancer patients, while it is rarely used to predict the expression of PD-L1 in NSCLC based on PET/CT images and clinicopathologic risk. Cancer cells within the same tumor are now recognized to be diverse in many ways. Many cell features, such as shape or phenotypic expression, display of inherent or acquired treatment resistance, and ability to initiate new tumor development, show heterogeneity. Intratumor heterogeneity is an important factor in determining tumor treatment response and patient prognosis (31). The blood perfusion, hypoxia, cell proliferation, necrosis, and other factors within the tumor cause significant internal biological differences (32). Because it gives an observer-independent measurement, SUV_max_ is a widely used parameter. MTV and TLG have been developed to measure the metabolic activity of the entire tumor mass. These parameters are designed to measure the overall changes in tumor glycolysis. Preliminary research has found that compared with SUV_max_ and SUV_mean_, ^18^F-FDG PET/CT image texture analysis can capture heterogeneity across the entire tumor and provide more valuable information in the diagnosis, staging, curative effect prediction, and prognosis of NSCLC (33). Preoperative SUV_max_ is correlated with PD-L1 expression in NSCLC patients. In the univariate analysis, the expression of PD-L1 in lung tumors was significantly correlated with SUV_max_, MTV, and TLG (p< 0.05). The features extracted from CT performed better than those of PET in assessing the expression status of PD-L1 both in the PD-L1 1% and 50% groups. The reason was attributed to that the density resolution of the PET image was not so good as the CT image, which could have a great effect on extracting and selecting the meaningful radiomic features. When combined with CT features, the model showed improvement in distinguishing the PD-L1 expression level between in the PD-L1 1% and 50% groups. A recent study has assessed the expression of PD-L1 by radiomic features from PET/CT images in NSCLC patients, showing that radiomic signatures of PD-L1 expression over 1% and 50% reach an AUC score of 0.85 and 0.880, respectively (34). However, they do not combine radiomic features with clinical risk factors in the prediction model. Sun *et al.* have assessed the expression of PD-L1 in tumor cells in NSCLC patients by using a radiomic study based on CT images and clinicopathologic features, and the score of AUC is 0.848 (35), which is consistent with our results. Another study has investigated the association between PD-L1 expression and textural features of PET images in 53 patients with oropharyngeal or hypopharyngeal cancer, while the sample number is too small, and the constructed prediction model of PD-L1 expression by the radiomics cannot be robust (36). A study has shown that PD-L1 is more common in patients with the following clinical characteristics: larger tumor size, more positive lymph node involvement, greater historically tumor grade, and higher Ki-67 index (37). Another study has pointed out that positive Ki-67 expression is strongly associated with positive PD-L1 expression (38), which is consistent with our results. In the present study, we classified NSCLC patients according to their PD-L1 expression levels and found that PD-L1 expression levels were associated with differences in gender, pathological type, and Ki-67 levels of patients. Wu *et al.* have found that the expression of PD-L1 is significantly associated with the advanced N stage but not with T and M stages (39). Subsequently, we incorporated the radiomic signature and clinicopathological factors into a combined model, which presented a better diagnostic efficacy (AUC=0.814) compared with the simple radiomic signature or clinical feature model both in the PD-L1 1% and 50% groups. In our present study, we found that for the prediction of PD-L1 expression over 1% and 50%, the AUC scores were 0.757 and 0.814 for features derived from the combined model, respectively. Our findings were consistent with the previous studies, indicating that PET radiomic features were useful to screen the NSCLC patients who could benefit from the anti-PD-L1 immunotherapy. However, only very few studies have investigated the sensitivity and specificity of PD-L1 in NSCLC patients. Many sources may cause these differences, such as small sample size, image segmentation, acquisition and reconstruction parameters, and feature extraction software. Further investigations in a larger cohort population are required to validate our conclusions.

Repeatability is a basic requirement in radiomic analysis (40, 41). In the present study, all ^18^F-FDG PET/CT images were realized in the same center using the same acquisition and reconstruction protocols. To reduce the impact of discretization values on robustness, a reliable discretization using a fixed size of bins was adopted (42, 43).

The present study has several limitations. First, this was a single-center retrospective study, and the sample size was small. This indicated that the variability among image characteristics from various localities was not completely captured, and potential selection bias might exist. Therefore, our results need to be confirmed by studies with larger sample sizes. Second, the expression of PD-L1 in the tumor had inherent instability in individual patients. Third, manual drawing ROI and manual image segmentation were adopted, which had poor reproducibility and high technical requirements for operators.

## Conclusions

In the present study, we established a prediction model based on PET/CT images and clinicopathological characteristics to predict the expression of PD-L1 in NSCLC patients and provided a novel strategy for clinicians to screen the patients who could benefit from anti-PD-L1 immunotherapy.

## Data Availability Statement

The original contributions presented in the study are included in the article/supplementary materials, further inquiries can be directed to the corresponding authors.

## Ethics Statement

Written informed consent was obtained from the individual(s) for the publication of any potentially identifiable images or data included in this article.

## Author Contributions

JL, SG, and SS contributed equally to this work. All authors contributed to the article and approved the submitted version.

## Funding

The present study was supported by the National Natural Science Foundation of China (grant no. 81601522), Medical Youth Talent Project of Jiangsu Province (grant no. QNRC2016749), Gusu Health Talent Program (grant no. GSWS2020013), Suzhou People’s Livelihood Science and Technology Project (grant no. SYS2019038), and Project of State Key Laboratory of Radiation Medicine and Protection, Soochow University, (No. GZK1202127).

## Conflict of Interest

The authors declare that the research was conducted in the absence of any commercial or financial relationships that could be construed as a potential conflict of interest.

The reviewer LZ declared a shared parent affiliation with the authors to the handling editor at the time of the review.

## Publisher’s Note

All claims expressed in this article are solely those of the authors and do not necessarily represent those of their affiliated organizations, or those of the publisher, the editors and the reviewers. Any product that may be evaluated in this article, or claim that may be made by its manufacturer, is not guaranteed or endorsed by the publisher.
